# Folic acid supplementation normalizes the endothelial progenitor cell transcriptome of patients with type 1 diabetes: a case-control pilot study

**DOI:** 10.1186/1475-2840-8-47

**Published:** 2009-08-25

**Authors:** Olivia van Oostrom, Dominique PV de Kleijn, Joost O Fledderus, Mario Pescatori, Andrew Stubbs, Attie Tuinenburg, Sai Kiang Lim, Marianne C Verhaar

**Affiliations:** 1 Department of Nephrology and Hypertension, University Medical Center Utrecht, Utrecht, the Netherlands; 2 Department of Experimental Cardiology, University Medical Center Utrecht, Utrecht, the Netherlands; 3 Interuniversity Cardiology Institute of the Netherlands, Utrecht, The Netherlands; 4 Department of Bioinformatics, Erasmus MC, Rotterdam, the Netherlands; 5 Institute of Medical Biology, A*STAR, Singapore

## Abstract

**Background:**

Endothelial progenitor cells play an important role in vascular wall repair. Patients with type 1 diabetes have reduced levels of endothelial progenitor cells of which their functional capacity is impaired. Reduced nitric oxide bioavailability and increased oxidative stress play a role in endothelial progenitor cell dysfunction in these patients. Folic acid, a B-vitamin with anti-oxidant properties, may be able to improve endothelial progenitor cell function. In this study, we investigated the gene expression profiles of endothelial progenitor cells from patients with type 1 diabetes compared to endothelial progenitor cells from healthy subjects. Furthermore, we studied the effect of folic acid on gene expression profiles of endothelial progenitor cells from patients with type 1 diabetes.

**Methods:**

We used microarray analysis to investigate the gene expression profiles of endothelial progenitor cells from type 1 diabetes patients before (n = 11) and after a four week period of folic acid supplementation (n = 10) compared to the gene expression profiles of endothelial progenitor cells from healthy subjects (n = 11). The probability of genes being differentially expressed among the classes was computed using a random-variance t-test. A multivariate permutation test was used to identify genes that were differentially expressed among the two classes. Functional classification of differentially expressed genes was performed using the biological process ontology in the Gene Ontology database.

**Results:**

Type 1 diabetes significantly modulated the expression of 1591 genes compared to healthy controls. These genes were found to be involved in processes regulating development, cell communication, cell adhesion and localization. After folic acid treatment, endothelial progenitor cell gene expression profiles from diabetic patients were similar to those from healthy controls. Genes that were normalized by folic acid played a prominent role in development, such as the transcription factors ID1 and MAFF. Few oxidative-stress related genes were affected by folic acid.

**Conclusion:**

Folic acid normalizes endothelial progenitor cell gene expression profiles of patients with type 1 diabetes. Signaling pathways modulated by folic acid may be potential therapeutic targets to improve endothelial progenitor cell function.

## Background

Diabetes mellitus (DM) is a major risk factor for micro- and macrovascular complications[1,2] and is associated with endothelial dysfunction, premature atherosclerosis [3-5], and a reduced capability of neovascularization in ischemic conditions[6]. Hyperglycemia increases the production of superoxide (O_2_^-^) and reduces the bioavailability of nitric oxide (NO) resulting in the development of endothelial dysfunction in diabetic patients[7,8]. Exposure to oxidative stress induces a pro-inflammatory response and increases endothelial cell apoptosis, which leads to a disturbance in the endothelial monolayer. The denuded vessel wall is highly pro-atherogenic, so fast regeneration of the endothelium is essential to prevent formation of atherosclerotic plaques[9,10].

Besides repair of the endothelial monolayer by adjacent mature endothelial cells, circulating bone marrow-derived endothelial progenitor cells (EPC) are also recognized to play an important role in reendothelialization [11-15]. Furthermore, studies have shown that *ex vivo *expanded EPC can home to sites of ischemia, express endothelial markers and improve neovascularization and tissue regeneration [16-18]. In addition, clinical trials are ongoing to evaluate the regenerative capacity of EPC in patients with ischemic limb or heart disease[19]. Patients with cardiovascular risk factors, such as type 1 or 2 DM, have decreased numbers of EPC and these show impaired functional capacity [20-24]. Mechanisms underlying endothelial dysfunction, such as reduced NO bioavailability and increased oxidative stress also play a role in EPC dysfunction in patients with DM[25,26].

Increasing NO bioavailability by improving endothelial nitric oxide synthase (eNOS) function can be achieved by folic acid (FA) supplementation. The active form of FA, 5-methyltetrahydrofolate restores the function of uncoupled eNOS by increasing the availability of its cofactor tetrahydrobiopterin (BH_4_)[27]. Studies have shown that FA supplementation restores endothelial function in patients with coronary artery disease[28,29], hyperhomocysteinemia [30-32], hypercholesterolemia [33-35], and type 1 and 2 DM [36-38]. Recently, it was shown that cardiac function can be preserved after ischemia in FA-treated rats[39]. In addition, exogenous BH_4_ improves pre-existing advanced cardiac hypertrophy and fibrosis in mice[40]. These studies[39,40] describe novel beneficial effects of FA, suggesting that its therapeutic potential in cardiovascular disease still remains to be fully elucidated. At the molecular level, the effects of DM on EPC are not well characterized. Although a role for oxidative stress in modulating EPC number and function has been implied[25,26], the effects of DM on EPC gene expression remain unclear. Autologous progenitor cell-based therapy may not reach its true potential in diabetic patients when their own progenitor cells are impaired. Therefore, a likely therapeutic strategy may be the modulation of EPC levels and/or function. Increased understanding of the mechanisms leading to the numerical and functional impairment of EPC is necessary. In this study, we investigated the gene expression profiles of EPC in DM type 1 (DM1) patients compared to healthy subjects. Furthermore, we show that FA can change the gene expression profiles of EPC from DM1 patients to resemble those of healthy subjects.

## Methods

### Subjects

Patients with DM1 (n = 20), diagnosed at least 1 year before entering the study, were recruited from the outpatient clinic of the Department of Internal Medicine of the University Medical Centre Utrecht, The Netherlands. Exclusion criteria were presence of manifest macrovascular disease, liver disease, homocysteine > 15 μmol/l, creatinine > 120 μmol/l and untreated thyroid disease. If patients were being treated with vasoactive medication (angiotensin converting enzyme inhibitors, angiotensin II antagonists, statins, nonsteroidal anti-inflammatory drugs (NSAIDs), FA or vitamins), treatment was stopped at least 3 weeks before initiation of the study. Twenty age- and gender-matched healthy subjects served as controls. Cardiovascular risk was evaluated by a questionnaire and clinical parameters such as weight, length and blood pressure were measured.

Peripheral blood samples were collected from 20 patients with DM1 and 20 age- and gender-matched healthy control subjects (CTR) at baseline. Patients with DM1 were then treated for 4 weeks with FA (Ratiopharm) 5 mg/day after which peripheral blood samples were collected again (19 of the 20 patients). The study protocol was approved by the Medical Ethical Committee of the University Medical Centre Utrecht. All participants in the study gave their written informed consent.

### EPC Culture

Peripheral blood samples (90 ml) were collected in EDTA tubes (Greiner Bio-One) and mononuclear cells (MNC) were isolated using Ficoll density gradient centrifugation (Ficoll-Paque Plus; GE Healthcare Bio-Sciences). MNC were plated on human fibronectin (Becton Dickinson)-coated six-well plates (Corning) at a density of 5 × 10^6 ^cells/ml of EGM-2 medium (Cambrex), supplemented with 20% fetal calf serum (Invitrogen), 100 ng/ml recombinant VEGF-165 (R&D systems) and antibiotics (penicillin 100 U/ml and streptomycin 100 μg/ml; Invitrogen). Mononuclear cells were kept in a stove at 37°C, for 7 days, allowing differentiation to EPC. At day 4, medium was changed to wash away non-adherent cells. Cells used for quantification were detached by using trypsin-EDTA (Invitrogen) and gentle cell scraping, followed by counting on a hemocytometer (Cell-Dyn 1800, Abbott Diagnostics). EPC were cultured as previously described[41]. EPC phenotype of attaching cells was confirmed by the presence of endothelial surface markers, the binding of Ulex Europaeus Agglutinin-1 and the uptake of DiI-labeled acetylated LDL.

### Microarray Analysis

Total RNA was extracted from Trizol^® ^(Invitrogen)-treated EPC samples from 20 DM1 patients before and after treatment with FA, and 20 age- and gender-matched healthy control subjects according to manufacturer's instructions. One sample from a FA treated DM1 patient was excluded due to low total RNA yield. Chromosomal DNA was removed from the samples by treatment with DNase I (Amersham Biosciences). The concentration of the isolated RNA was determined with the Nanodrop ND-1000 spectrophotometer (Nanodrop Technologies). The samples were independent isolates from single donors.

Microarray analysis was performed on EPC samples from a subset of the patient population i.e. 11 DM1 patients, 10 DM1 patients after FA and 11 healthy controls. Double-stranded cDNA was synthesized from 125 ng of total RNA and in vitro transcription was performed to generate biotinylated cRNA using the Illumina^® ^TotalPrep™ RNA Amplification Kit (Ambion) according to the manufacturer's instructions. From each sample, 850 ng of cRNA was hybridized overnight at 55°C to Sentrix HumanRef-8 Expression BeadChips (Illumina), containing ~23000 genes. The following day the BeadChips were washed and a signal was developed with streptavidin-Cy3 (Amersham Biosciences). Chips were scanned with a BeadArray Reader (Illumina).

Raw gene array bead summary intensities were extracted using Beadstudio version 3.2 (Illumina) and quantile normalized in R/Bioconductor. Normalized bead summary intensities were imported in BRB-ArrayTools (developed by Dr. Richard Simon and BRB-ArrayTools Development Team) for further analysis. Microarray data are available at , accession number GSE17635.

### Statistical Analysis

Differences in EPC numbers between patients with DM1 and healthy controls, and patients with DM1 before and after FA treatment were determined by a Mann Whitney or Wilcoxon signed rank test respectively. A p value < 0.05 was considered statistically significant.

The probability of genes being differentially expressed among the classes was computed using a random-variance t-test. The random-variance t-test is an improvement over the standard separate t-test as it permits sharing information among genes about within-class variation without assuming that all genes have the same variance[42], A multivariate permutation test was used to identify genes that were differentially expressed among the two classes[43,44]. The multivariate permutation test provides 90% confidence that the false discovery rate (FDR) is less than 10%. The test statistics used are random variance t-statistics for each gene[42]. Although t-statistics were used, the multivariate permutation test is non-parametric and does not require the assumption of Gaussian distributions. Gene clusters with similar gene expression patterns across sample classes were identified by using hierarchical clustering (Pearson *1 – correlation *distances) on normalized bead signal intensities. Functional classification of differentially expressed genes was performed using the biological process ontology in the Gene Ontology database (GO; ). Classification was listed if a biological process term was significantly overrepresented or underrepresented among differentially expressed genes as compared to the Homo sapiens reference genome.

## Results

### Patient Characteristics

Patient characteristics are summarized in Table 1. The group of diabetic patients is representative of a type 1 diabetic population without macrovascular complications. Retinopathy was present in 4 patients. None of the patients had microalbuminuria. Antihypertensive drugs, statins and FA supplementation were stopped at least 3 weeks before initiation of the study. Age and gender-matched healthy controls did not significantly differ in body mass index and blood pressure from patients with DM1. FA treatment did not have any effect on the characteristics.

**Table 1:** Characteristics of patients with DM1. Data are mean ± SEM.

	Type 1 diabetic patients (n = 20)
Age (years)	34.2 ± 6.4
Gender (male/female)	8/12
Body mass index (kg/m^2^)	22.8 ± 1.7
Blood pressure (mmHg)	
Systolic	128 ± 13
Diastolic	83 ± 8
Duration of diabetes (years)	14.0 ± 6.6
Medication	
Insulin	20/20
Antihypertensive drugs	2/20
NSAIDs	1/20
Statin	1/20
Oral anti conception	8/12
Folic acid	3/20
Current smoker	6/20
Glucose (mmol/l)	8.4 ± 3.6
HbA1c (%)	8.2 ± 0.6
Homocysteine (μmol/l)	8.2 ± 2.5
Total cholesterol (mmol/l)	4.3 ± 0.6
LDL (mmol/l)	2.3 ± 0.5
HDL (mmol/l)	1.6 ± 0.2
Triglycerides (mmol/l)	0.9 ± 0.3

### Effect of DM1 on EPC Number

**Figure 1:**
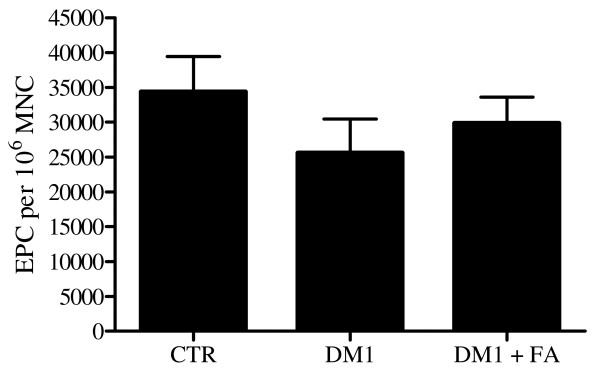
**Effect of DM1 on EPC number**. EPC number from diabetic patients before (DM1, n = 20) and after treatment with FA (DM1 + FA, n = 19) and from age- and gender-matched healthy control subjects (CTR, n = 20). Data are presented as mean ± SEM.

We assessed the number of EPC obtained after 7-day culturing of peripheral blood MNC from patients with DM1 before (n = 20) and after 4 weeks (n = 19) of treatment with FA and from healthy control subjects (n = 20). We observed a 26% decrease in the mean number of EPC from DM1 patients compared to healthy control subjects (25575 ± 4891 versus 34375 ± 5065 EPC/10^6 ^MNC; p = 0.057). After FA treatment, the mean number of EPC tended to increase slightly by 17% (25575 ± 4891 versus 29868 ± 3754 EPC/10^6 ^MNC; p = 0.14) (Figure 1). No significant differences in EPC number were observed between healthy controls and DM1 patients after FA (34375 ± 5065 versus 29868 ± 3754 EPC/10^6 ^MNC; p = 0.37).

### Differential Gene Expression in EPC from Patients With DM1 Before and After FA Treatment and Healthy Controls

**Figure 2:**
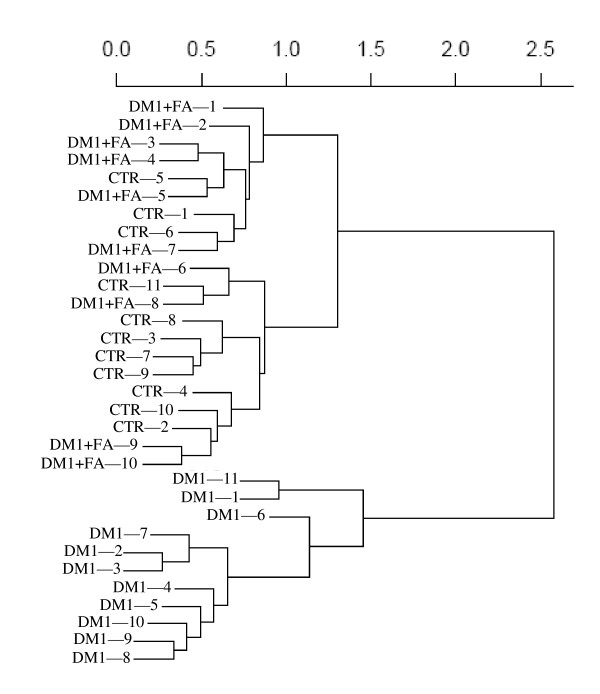
**Similarity of gene expression profiles of EPC between healthy controls and FA-treated DM1 patients**. Unsupervised hierarchical clustering based on gene expression of EPC from DM1 patients before and after FA treatment and healthy controls. Clustering of conditions partitioned samples into 2 groups. The gene expression profiles of healthy controls (CTR) and FA-treated DM1 patients are clustered together as a distinct group, separated from the gene expression profiles of EPC from patients with DM1 before FA-treatment.

Whole genome microarray analysis was performed to compare gene expression profiles of EPC from patients with DM1 before and after FA treatment and healthy controls. At a FDR of 0.05, a total of 2170 genes were differentially expressed between DM1 patients before and after FA treatment and healthy controls. This set of genes was used for hierarchical clustering analysis. The dendrogram in Figure 2 demonstrates the close correlation of the gene expression profiles of EPC from FA-treated DM1 patients and healthy controls which are clustered together while gene expression profiles of EPC from DM1 patients are placed in a distinct group.

**Figure 3:**
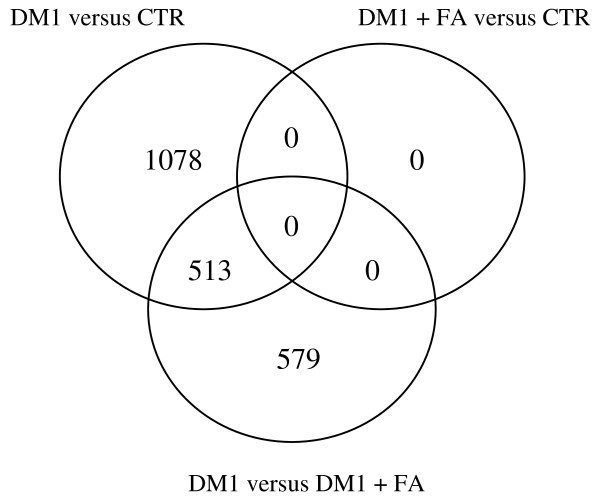
**A Venn diagram depicting differential gene expression**. The distribution is represented of 2170 probe sets found to be differentially expressed in EPC in at least 1 comparison (DM1, DM1 + FA, CTR) at a FDR of 0.05.

The result of the differential gene expression analysis is summarized in Figure 3 with the Venn diagram depicting the distribution of 2170 probe sets found to be differentially expressed in EPC in at least 1 comparison at a FDR of 0.05. 1591 genes are differentially expressed between DM1 patients and healthy controls, and 1092 genes are differentially expressed in DM1 patients before and after FA. The intersect shows that 513 genes with differential expression between healthy controls and DM1 are also modulated by FA treatment. Gene expression profiles of EPC from healthy controls and DM1 patients after FA were very similar and pairwise comparison shows no differentially expressed genes at a 0.05 FDR level.

### Classification of Differentially Expressed Genes in EPC of Patients With DM1 Compared to Healthy Controls

To investigate the effect of DM1 on EPC gene expression on a more functional level, differentially regulated genes in EPC of patients with DM1 compared to healthy controls were classified into Gene Ontology (GO) terms (Table 2). Fifty-five GO terms with a p value of less than 0.01 in the differentially expressed gene set were enriched compared to a random gene set of equal size, resulting in an enrichment including 'cell communication', 'developmental process', 'localization', 'cell proliferation' and 'cell adhesion'. 30% of the differentially regulated genes were involved in cell communication, including members of the desintegrin and metalloproteinase domain family (upregulated HOXD9, ID1, NFATC3; downregulated MAFF, HOXB8, HOXD8), interleukin family (upregulated IL12RB1, IL12RB2, IL13, IL18, IL1RAP; downregulated IL1RL1, IL9R), RAS GTPase superfamily (upregulated RAB39B, RABL2A, RASAL1, RASAL2, and RASD2; downregulated RAB11B, RAB27A, RAB3B, RABL4, RASA1) and TNF receptor family (downregulated TNFRSF-14, -17). 26% of the genes were categorized into the GO term 'developmental process', including members of the fibroblast growth factors (upregulated FGF3, FGF11), transcription regulators (upregulated HOXD9, ID1, NFATC3; downregulated MAFF, HOXB8, HOXD8), and members of the wingless-type (Wnt) pathway (upregulated FRZB, WNT10B, WNT11).

**Table 2:** Classification of differentially expressed genes between healthy controls and DM1 patients according to gene ontology (GO) terms with a p value < 0.01.

**Term**	**Category**	**% of genes in category**	**p value**
GO:0032501	multicellular organismal process	32,02	9,28E-09
GO:0007275	multicellular organismal development	21,45	1,93E-07
GO:0048856	anatomical structure development	19,24	3,11E-06
GO:0007267	cell-cell signaling	7,89	5,22E-06
GO:0006812	cation transport	6,94	5,94E-06
GO:0048731	system development	16,25	6,22E-06
GO:0032502	developmental process	26,18	1,94E-05
GO:0006811	ion transport	8,99	2,27E-05
GO:0030001	metal ion transport	5,52	7,57E-05
GO:0009605	response to external stimulus	7,10	1,11E-04
GO:0015672	monovalent inorganic cation transport	4,57	1,19E-04
GO:0048513	organ development	11,83	1,56E-04
GO:0006813	potassium ion transport	2,84	2,01E-04
GO:0051179	localization	24,29	4,04E-04
GO:0006091	generation of precursor metabolites and energy	6,78	4,90E-04
GO:0007167	enzyme linked receptor protein signaling pathway	3,79	5,36E-04
GO:0003012	muscle system process	2,68	6,04E-04
GO:0006936	muscle contraction	2,68	6,04E-04
GO:0019226	transmission of nerve impulse	4,26	7,76E-04
GO:0007188	G-protein signaling, coupled to cAMP nucleotide second messenger	1,74	1,35E-03
GO:0008283	cell proliferation	7,57	1,65E-03
GO:0006950	response to stress	9,62	1,76E-03
GO:0008284	positive regulation of cell proliferation	3,15	1,97E-03
GO:0009611	response to wounding	4,73	1,99E-03
GO:0007189	G-protein signaling, adenylate cyclase activating pathway	1,10	2,11E-03
GO:0019933	cAMP-mediated signaling	1,74	2,27E-03
GO:0006954	inflammatory response	3,63	2,53E-03
GO:0007154	cell communication	29,50	2,75E-03
GO:0007155	cell adhesion	7,26	2,76E-03
GO:0022610	biological adhesion	7,26	2,76E-03
GO:0006928	cell motility	4,57	2,81E-03
GO:0051674	localization of cell	4,57	2,81E-03
GO:0030041	actin filament polymerization	0,95	3,08E-03
GO:0044262	cellular carbohydrate metabolic process	3,94	3,72E-03
GO:0007156	homophilic cell adhesion	2,21	3,85E-03
GO:0006952	defense response	5,68	4,07E-03
GO:0031279	regulation of cyclase activity	1,10	4,16E-03
GO:0051339	regulation of lyase activity	1,10	4,16E-03
GO:0007187	G-protein signaling, coupled to cyclic nucleotide second messenger	1,89	4,78E-03
GO:0016051	carbohydrate biosynthetic process	1,89	4,78E-03
GO:0019317	fucose catabolic process	0,63	5,29E-03
GO:0042355	L-fucose catabolic process	0,63	5,29E-03
GO:0007173	epidermal growth factor receptor signaling pathway	0,95	5,72E-03
GO:0007268	synaptic transmission	3,47	5,92E-03
GO:0001501	skeletal development	2,84	6,00E-03
GO:0008154	actin polymerization and/or depolymerization	1,26	6,04E-03
GO:0007610	behavior	3,63	6,90E-03
GO:0007169	transmembrane receptor protein tyrosine kinase signaling pathway	2,52	6,99E-03
GO:0019935	cyclic-nucleotide-mediated signaling	1,89	7,81E-03
GO:0003008	system process	11,67	8,19E-03
GO:0007626	locomotory behavior	2,52	8,37E-03
GO:0042127	regulation of cell proliferation	4,89	8,47E-03
GO:0001666	response to hypoxia	1,10	9,08E-03
GO:0042354	L-fucose metabolic process	0,63	9,17E-03
GO:0043085	positive regulation of catalytic activity	2,84	9,54E-03

Cell adhesion plays an important role in progenitor cell biology and has previously been shown to be impaired in EPC from DM patients[24]. We therefore examined genes that were affected in our DM1 population and could potentially have a role in EPC adhesion. We found that members of the protocadherin family involved in calcium-dependent cell adhesion (upregulated PCDH8, PCDHB8, PCDHB9, PCDHGA4, PCDHGB3; downregulated PCDHA8, PCDHGA10, PCDHGA3, PCDHGA7, PCDHGB2), structural proteins (upregulated SPON1, COL13A1; downregulated COL21A1) and integrin-related binding proteins (upregulated ADAM22, IBSP, ITGB1; downregulated ADAM2) were included in the category 'cell adhesion'.

We also examined the genes categorized into GO terms 'response to stress' and 'response to hypoxia' because of the known effect of diabetes on oxidative stress-related signaling. These genes included dual oxidase 2 (DUOX2), which is a NADPH oxidase and has the capacity to generate superoxide, nitric oxide synthase 2A (NOS2A), which can produce nitric oxide, thioredoxin reductase 2 (TXNRD2), a key enzyme in the regulation of the intracellular redox balance, lactoperoxidase (LPO) and NADPH oxidase organizer 1 (NOXO1), which is involved in reactive oxygen species production, all of which were upregulated in EPC from patients with DM1.

### FA Normalizes Changes in Gene Expression in EPC From Patients With DM1

**Figure 4:**
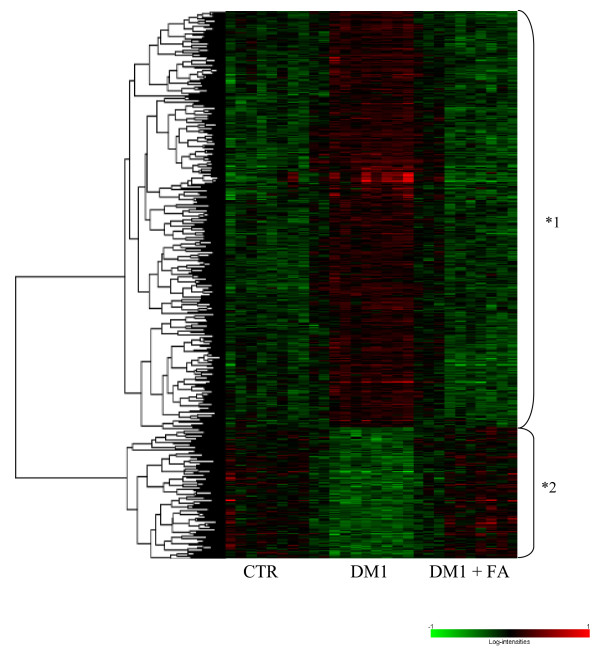
**Heat map display of genes that are normalized by FA in EPC of DM1 patients**. Visualization of mean-centered and normalized gene expression patterns of the 513 intersect genes with differential expression between healthy controls (CTR) and DM1 patients that are normalized by FA treatment. The relative levels of gene expression are depicted with a color scale, where green represents the lowest and red the highest level of expression. Two main clusters of genes can be clearly identified as marked by an asterisk.

Interestingly, 513 genes that were differentially expressed between healthy controls and patients with DM1 were normalized by FA treatment. Genes with this expression pattern are visualized in the heat map depicted in Figure 4. 390 genes were upregulated (cluster 1) and 123 genes (cluster 2) were downregulated in DM1 (for the complete list of gene names, see Additional file 1) and the expression of these genes was normalized to healthy control levels after FA treatment.

**Figure 5:**
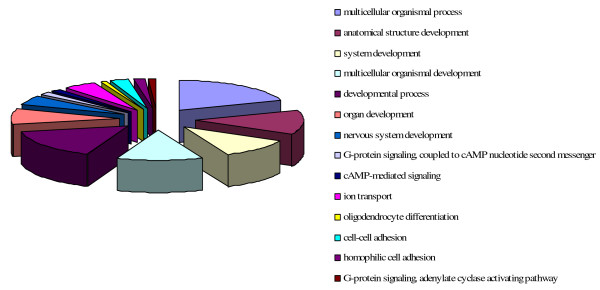
**A pie chart representing classification of 513 genes into Gene Ontology terms**. Biological processes, defined by genes that are normalized by FA treatment in EPC from patients with DM1, with a p value < 0.01 are listed.

To more closely examine the observed effects of FA on diabetic EPC gene expression, we classified the 513 genes into Gene Ontology terms (Figure 5). Fourteen terms in the list had a p value of less than 0.01. Strikingly, many of these processes are related to development, indicating a major effect of FA on developmental processes. Gene expression of fibroblast growth factor 3 (FGF3), transcriptional regulators (HOXD8, HOXD9, ID1, MAFF), and members of the Wnt pathway (FRZB, WNT11) was significantly different between DM1 and healthy controls and normalized by FA. We also examined the category 'cell adhesion', which was modulated by DM1 and found that FA regulated several members of the protocadherin family (PCDH8, PCDHB8, PCDHB9, PCDHGA10, PCDHGA4, PCDHGB3), structural protein collagen type XIII alpha 1 (COL13A1) and integrin beta 1 (ITGB1). Furthermore, genes involved in G protein signaling including guanine nucleotide binding proteins (GNAL, GNAQ), cortistatin (CORT), gamma-aminobutyric acid B receptor 1 (GABBR1), gastric inhibitory polypeptide receptor (GIPR) and parathyroid hormone-like hormone (PTHLH) were regulated by FA. Four oxidative stress-related genes (DUOX2, NOS2A, NOXO1 and LPO) which were differentially expressed between DM1 and healthy controls returned to control levels by FA.

## Discussion

In this study we investigated the effects of FA on the transcriptome of EPC from patients with DM1. We found that DM1, compared to healthy controls, modulates the expression of 1591 genes which are mainly involved in cell communication, development, localization, cell proliferation and cell adhesion. FA treatment for 4 weeks in patients with DM1 leads to the normalization of the gene expression of 513 of the 1591 genes. In particular, FA regulated genes that are involved in development, cell adhesion and G protein signaling.

This study describes differential gene expression profiling of EPC between patients with DM1 and healthy controls. Importantly, in order to be able to improve impaired numerical and functional capacity of EPC, identification of genes that are affected by DM1 is required. We observed an effect of DM1 on members of the desintegrin and metalloproteinase domain family (ADAM17, ADAM22, ADAMTS14 and ADAMTS18) involved in cell communication. ADAM22, a membrane spanning protein, is one of the most strongly upregulated genes in DM1 compared to healthy controls. Little is known about this member of the ADAM family, however, it has been shown that other members such as ADAM17 can potentially play a role in angiogenesis and cytokine biology in inflammatory processes[45,46]. Of note, ADAM17, also known as TNF-α converting enzyme which is important in processing and release of TNF-α from the cell membrane, was significantly upregulated in EPC of DM1 patients[45,46].

Genes that also play a role in cell communication and development are members of the TNF receptor family (TNFRSF-14, and -17). TNFRSF-14 was significantly downregulated in EPC of DM1 patients compared to healthy controls. It has been described that this receptor can play a dual role in regulating T cell immune responses depending on ligand receptor interaction. Knockout animals are more susceptible to developing autoimmune diseases[47], and T cell survival depends on receptor activation[48]. In contrast, the receptor has been implicated to play a role in inflammatory diseases such as rheumatoid arthritis[49,50] and atherosclerosis[51]. So far, no studies have reported TNFRSF-14 expression in EPC, therefore it remains to be investigated what its potential role could be in EPC function.

Other groups have performed microarray analysis on circulating cells from patients with DM1, in particular monocytes[52] and peripheral blood mononuclear cells[53]. As EPC are cultured from the peripheral blood mononuclear cell fraction and have been previously described as a monocyte-like cell[54], some overlap in gene expression between the different cell types can be expected. Interestingly, similar expression patterns were seen in EPC from DM1 patients in our study, and monocytes and peripheral blood mononuclear cells from DM1 patients in the other studies[52,53], for splicing factor, arginine/serine rich 15 (SFRS15), cell division cycle 42 (CDC42), interleukin 6 (IL6), heat shock 70 kDa protein 1A (HSPA1A), and chemokine ligand 20 (CCL20).

As expected, DM1 affected oxidative stress-related processes in comparison with healthy controls. In particular, we found that expression of DUOX2, NOS2A, TXNRD2, LPO and NOXO1 was upregulated in DM1 patients. FA exerts protective anti-oxidant effects and has shown to improve endothelial function in patients with cardiovascular disease [28-38], In our study, we expected an effect of FA treatment on the expression of oxidative stress-related genes in EPC. FA indeed regulated expression of DUOX2, NOS2A, NOXO1 and LPO. Unexpectedly, we identified a striking effect of FA on genes involved in developmental processes, cell adhesion and G protein signaling. Inhibitor of differentiation 1 (ID1) was upregulated in DM1 and normalized by FA. ID1 is a basic helix-loop-helix transcription factor that lacks a DNA binding domain. Through its ability to bind to the ubiquitously expressed E protein family of basic helix-loop-helix transcription factors, it can inhibit their binding to target DNA. This important function of Id proteins confers a central role in the regulation of gene expression and hence cellular differentiation and proliferation[55,56]. Interestingly, Id knockout mice show a complete loss of EPC in the peripheral blood, which is correlated with a block in tumor neovascularization and delayed tumor growth[57].

V-maf musculoaponeurotic fibrosarcoma oncogene homolog F (MAFF) is a transcription factor that was downregulated in DM1 and normalized by FA. Although little is known about the functional role of MAFF in health and disease, some studies have shown that it plays a role in the cellular stress response. MAFF can bind to Nrf2, a transcription factor that activates the expression of genes with anti-oxidant response elements in their promoters[58,59]. This is the first time that MAFF has been described in association with DM1.

Moens et al[40] recently showed that exogenous BH4 could reverse pre-existing cardiac fibrosis and hypertrophy in mice. A microarray analysis was performed to identify genes that are regulated by BH4 in comparison to another anti-oxidant approach (Tempol). It was reported that BH4 had an effect on expression of ATP- and metabolic and G protein signaling-regulated genes. This indicates that FA may possess a more general modifying role in pathological processes besides its known capacity to restore NOS coupling.

We observed a decrease in EPC number in patients with DM1 compared to healthy controls consistent with other reports[21,24]. Treatment with FA only corrected for a small part the number of EPC in patients with DM1 which might be through the observed effect on ID1, modulating differentiation and proliferation, or apoptosis[60].

Further research will be needed to more closely investigate the individual role that the genes present in the set of FA-regulated genes in EPC function. Signaling pathways modulated by folic acid may be potential therapeutic targets to improve endothelial progenitor cell function.

## Conclusion

This study employed genome-wide profiling to elucidate the effect of DM1 and the response to FA on EPC gene expression. We observed a remarkable effect of FA on gene expression, resulting in normalization of EPC from DM1 patients to resemble those of healthy controls.

## Competing interests

The authors declare that they have no competing interests.

## Authors' contributions

OVO participated in the design of the study, collected the material, performed the microarray and analysis of the data, drafted and revised the manuscript. DPVDK participated in the analysis of the study and helped draft the manuscript. JOF participated in the analysis of the study and helped draft the manuscript. MP participated in interpretation and analysis of data, and revised the content critically for important intellectual content. AS participated in interpretation and analysis of data, and revised the content critically for important intellectual content. AT participated in interpretation and analysis of data, and revised the content critically for important intellectual content. SKL participated in the microarray analysis, and revised the content critically for important intellectual content. MCV participated in the design of the study, interpretation and analysis of data, and helped draft and revise the manuscript. All authors read and approved the final manuscript.

## Supplementary Material

Additional file 1 **Gene list for cluster 1 and 2**. Gene lists for clusters 1 and 2 are provided, including their fold changes and FDR. These genes are differentially expressed in EPC between DM1 and healthy controls and are normalized by FA treatment. Click here for file
