# Supplementary material for: Folic acid supplementation normalizes the endothelial progenitor cell transcriptome of patients with type 1 diabetes: a case-control pilot study
Source: Cardiovasc Diabetol. 2009 Aug 25;8:47. doi: 10.1186/1475-2840-8-47 (PMC2739843; doi:10.1186/1475-2840-8-47)
Supplement: Additional file 1 — Gene list for cluster 1 and 2. Gene lists for clusters 1 and 2 are provided, including their fold changes and FDR. These genes are differentially expressed in EPC between DM1 and healthy controls and are normalized by FA treatment. [file 1475-2840-8-47-S1.pdf]

	Gene symbol	Illumina Target ID	Fold change DM1/CTR	FDR	Fold change DM1+FA/CTR	FDR
cluster 1	DKFZp547G0215	GI_27735064-S	1,28	< 1e-07	1,09	2,21E-01
	GNB5	GI_20336269-I	1,29	< 1e-07	1,09	2,21E-01
	LOC401295	GI_42657675-S	1,25	< 1e-07	1,05	7,55E-01
	LOC393037	GI_41148356-S	1,25	1,71E-05	1,07	2,21E-01
	ADRA1A	GI_15451758-I	1,20	1,92E-05	1,07	2,85E-01
	HAPLN4	GI_30794471-S	1,19	1,92E-05	1,02	9,09E-01
	LOC401256	GI_42657630-S	1,17	2,28E-05	1,08	2,21E-01
	FLJ43806	GI_42491369-S	1,27	2,56E-05	1,08	7,55E-01
	HIST1H2BO	GI_16306565-S	1,22	2,80E-05	1,04	8,57E-01
	GNAT1	GI_22027519-A	1,19	2,99E-05	1,04	8,57E-01
	ID1	GI_31317296-I	1,22	3,02E-05	1,06	7,55E-01
	KRN1	GI_40254818-S	1,24	3,02E-05	1,01	9,71E-01
	LOC285941	GI_42658244-S	1,30	3,02E-05	1,11	2,21E-01
	TCF2	GI_6031204-I	1,23	3,02E-05	1,10	2,21E-01
	ZNF278	GI_14670359-I	1,21	3,02E-05	1,05	7,90E-01
	KRT9	GI_4557704-S	1,22	3,14E-05	1,02	9,46E-01
	LOC221061	GI_37551029-S	1,20	4,15E-05	1,01	9,71E-01
	LOC378136	GI_37539093-S	1,22	4,15E-05	1,09	3,16E-01
	OR1D5	GI_7657422-S	1,17	4,15E-05	1,07	3,58E-01
	ADAM22	GI_21536384-A	1,22	4,18E-05	1,02	9,71E-01
	DKFZp547I014	GI_9910199-S	1,16	4,18E-05	1,04	7,55E-01
	GNAL	GI_4504042-A	1,20	4,18E-05	1,01	9,71E-01
	LOC377739	GI_37540349-S	1,14	4,18E-05	0,98	9,05E-01
	LOC402469	GI_42658240-S	1,24	4,18E-05	1,04	9,10E-01
	SERPINC1	GI_4502260-S	1,15	4,18E-05	1,01	9,71E-01
	SYNE2	GI_33624860-I	1,27	4,95E-05	1,12	2,21E-01
	KIAA1937	GI_37549525-S	1,26	5,30E-05	1,03	9,04E-01
	LOC375533	GI_37551146-S	1,22	5,55E-05	1,04	8,80E-01
	LOC400517	GI_42660934-S	1,15	5,55E-05	1,05	7,55E-01
	LOC401451	GI_42658882-S	1,19	5,55E-05	1,06	7,55E-01
	MAGEA2b	GI_34335237-S	1,18	5,55E-05	0,99	9,71E-01
	OR2S2	GI_10092668-S	1,24	5,55E-05	1,06	7,55E-01
	RORA	GI_19743902-I	1,19	5,55E-05	1,01	9,71E-01

SLC38A3	GI_40795668-S	1,20	5,55E-05	1,03	8,97E-01
DDC	GI_4503280-S	1,20	6,45E-05	1,04	8,03E-01
B3GALT5	GI_15451886-I	1,25	7,14E-05	1,07	7,55E-01
CYP26A1	GI_16933529-I	1,16	7,14E-05	1,00	9,71E-01
CYP8B1	GI_13929428-S	1,15	7,14E-05	1,04	7,97E-01
KCNS2	GI_45433545-S	1,20	7,14E-05	1,00	9,88E-01
PITPNM2	GI_24308236-S	1,16	7,14E-05	1,04	8,57E-01
RAMP3	GI_5032022-S	1,18	7,14E-05	1,02	9,34E-01
RHBDL4	GI_21264325-S	1,24	7,14E-05	1,04	8,82E-01
LOC401939	GI_42656018-S	1,11	7,20E-05	1,01	9,34E-01
BACH	GI_32528285-I	1,18	7,59E-05	1,00	9,71E-01
LOC401032	GI_42656581-S	1,21	7,75E-05	0,98	9,61E-01
LOC135924	GI_37538531-S	1,18	7,80E-05	1,07	7,55E-01
RASAL2	GI_25121934-A	1,18	7,85E-05	1,03	8,97E-01
KIAA0703	GI_7662255-S	1,15	8,36E-05	1,04	7,55E-01
SLC6A3	GI_38194225-S	1,21	8,36E-05	1,03	9,49E-01
UGT1A8	GI_31377617-S	1,19	8,36E-05	1,03	9,34E-01
CHAT	GI_11038626-I	1,24	8,86E-05	1,01	9,71E-01
PCDHGA4	GI_14196467-A	1,13	9,80E-05	1,01	9,71E-01
LOC127309	GI_37548619-S	1,28	1,00E-04	1,09	7,55E-01
LOC153441	GI_27481869-S	1,17	1,00E-04	1,02	9,34E-01
LOC254897	GI_37548610-S	1,22	1,00E-04	1,04	8,79E-01
LOC255244	GI_22064848-S	1,25	1,00E-04	1,11	2,75E-01
LOC374493	GI_37545362-S	1,18	1,00E-04	1,04	8,57E-01
LPO	GI_40549417-S	1,22	1,00E-04	1,04	8,97E-01
PACRG	GI_22748868-S	1,18	1,00E-04	1,04	8,57E-01
PGR6	GI_37547343-S	1,21	1,00E-04	1,04	8,79E-01
LOC401100	GI_42656878-S	1,21	1,02E-04	1,06	8,57E-01
DMRTB1	GI_24308351-S	1,18	1,09E-04	1,03	9,32E-01
FLJ90834	GI_27734820-S	1,30	1,09E-04	1,07	7,55E-01
LOC375457	GI_37550371-S	1,19	1,09E-04	1,02	9,71E-01
MGC27019	GI_34222169-S	1,26	1,09E-04	1,07	7,90E-01
PTHLH	GI_39995087-S	1,28	1,09E-04	1,07	7,97E-01
SLC22A1	GI_23510408-I	1,21	1,09E-04	1,06	7,84E-01
ZNF585A	GI_40217797-S	1,33	1,09E-04	1,09	7,55E-01
CDH3	GI_45269142-S	1,30	1,11E-04	1,05	8,57E-01

FGF3	GI_15451899-S	1,21	1,11E-04	1,08	2,21E-01
FLJ36561	GI_32698877-S	1,25	1,11E-04	1,05	8,57E-01
LOC377697	GI_37555920-S	1,21	1,11E-04	1,00	9,88E-01
PLAT	GI_14702166-A	1,18	1,11E-04	1,06	7,55E-01
LOC387765	GI_41200910-S	1,13	1,14E-04	1,02	8,79E-01
FLJ37357	GI_27735076-S	1,25	1,15E-04	1,05	8,79E-01
FLJ40243	GI_38564324-S	1,17	1,15E-04	1,06	7,55E-01
RPS4L	GI_17484248-S	1,19	1,15E-04	0,95	8,57E-01
TFAP2BL1	GI_27363485-S	1,25	1,15E-04	1,03	9,34E-01
ZFYVE27	GI_34303918-S	1,24	1,15E-04	1,05	8,57E-01
FLJ35794	GI_31377565-S	1,17	1,16E-04	1,01	9,71E-01
LOC254109	GI_37545358-S	1,15	1,18E-04	1,01	9,71E-01
ARTN	GI_16950639-I	1,27	1,19E-04	1,02	9,54E-01
PPP1R1B	GI_21735491-I	1,14	1,26E-04	1,00	9,77E-01
SPRL4A	GI_30387641-S	1,25	1,26E-04	1,03	8,79E-01
KIAA0265	GI_44917618-S	1,22	1,29E-04	1,06	7,97E-01
CAMTA2	GI_29826340-S	1,24	1,31E-04	1,01	9,71E-01
FIS	GI_31342253-S	1,24	1,31E-04	1,07	7,97E-01
LOC400047	GI_42659974-S	1,18	1,37E-04	0,98	9,49E-01
MLLT6	GI_5174576-S	1,13	1,37E-04	0,98	9,49E-01
PTPRH	GI_4506312-S	1,24	1,47E-04	1,01	9,71E-01
LOC151451	GI_18553104-S	1,24	1,54E-04	1,06	8,57E-01
ADORA1	GI_4501946-S	1,16	1,60E-04	1,05	7,55E-01
DIABLO	GI_42544196-A	1,49	1,68E-04	1,16	7,55E-01
PLUNC	GI_18765703-A	1,27	1,75E-04	1,04	8,97E-01
VN1R1	GI_18105064-S	1,17	1,75E-04	1,01	9,71E-01
GALNT5	GI_32698685-S	1,19	1,84E-04	0,99	9,71E-01
LOC219858	GI_20558728-S	1,22	1,87E-04	1,03	9,34E-01
FLJ37396	GI_27734722-S	1,53	1,92E-04	1,05	9,05E-01
LOC283816	GI_37541417-S	1,21	1,93E-04	1,05	8,57E-01
SLA/LP	GI_24432103-I	1,26	1,98E-04	0,99	9,71E-01
FLJ20519	GI_34147608-S	1,17	1,99E-04	1,06	7,55E-01
ARHGAP8	GI_31317213-I	1,23	2,03E-04	1,02	9,71E-01
B3GALT1	GI_15451870-S	1,25	2,03E-04	0,99	9,71E-01
DSCR10	GI_22380642-S	1,21	2,03E-04	1,03	9,34E-01
RNF129	GI_37622897-S	1,18	2,03E-04	0,98	9,46E-01

AKT2	GI_6715585-S	1,18	2,06E-04	1,04	8,57E-01
KIAA0889	GI_23308686-I	1,24	2,06E-04	1,01	9,71E-01
PRKAB2	GI_40254820-S	1,25	2,06E-04	1,06	8,57E-01
LOC283604	GI_37546007-S	1,17	2,07E-04	1,01	9,71E-01
APOBEC3C	GI_22907038-S	1,26	2,12E-04	0,98	9,71E-01
GABBR1	GI_11497611-I	1,27	2,12E-04	1,06	8,57E-01
NOXO1	GI_34222190-S	1,19	2,12E-04	1,00	9,82E-01
SPAG8	GI_27502372-A	1,30	2,12E-04	1,01	9,71E-01
ITIH3	GI_10092578-S	1,17	2,17E-04	1,01	9,71E-01
PDLIM1	GI_20127594-S	1,32	2,21E-04	1,02	9,71E-01
FLJ13769	GI_13376532-S	1,88	2,21E-04	1,16	8,03E-01
FLJ34907	GI_29743984-S	1,15	2,21E-04	1,02	9,16E-01
LOC377169	GI_37540377-S	1,17	2,21E-04	1,00	9,87E-01
STARD6	GI_21040260-S	1,16	2,41E-04	1,04	7,97E-01
CYP20A1	GI_29171729-I	1,22	2,50E-04	1,05	8,57E-01
KRT8	GI_4504918-S	1,23	2,59E-04	1,01	9,71E-01
VGLL1	GI_31543932-S	1,20	2,59E-04	1,05	8,57E-01
FLJ36004	GI_22749214-S	1,19	2,75E-04	1,05	7,97E-01
DUOX2	GI_28872752-S	1,27	2,76E-04	1,08	8,57E-01
FLJ90165	GI_23503264-S	1,13	2,76E-04	1,02	8,97E-01
LOC219870	GI_20558763-S	1,18	2,76E-04	1,06	7,55E-01
LOC376583	GI_37547270-S	1,19	2,76E-04	0,97	9,18E-01
CACNG1	GI_22027542-S	1,16	2,76E-04	0,99	9,71E-01
IRX2	GI_39930474-S	1,17	2,78E-04	0,99	9,71E-01
KIAA1110	GI_42660071-S	1,17	2,78E-04	1,02	9,57E-01
SYT7	GI_38570145-S	1,14	2,81E-04	0,97	8,79E-01
ISL1	GI_4504736-S	1,22	2,84E-04	1,00	9,82E-01
PSCA	GI_29893565-S	1,16	2,87E-04	1,05	7,55E-01
PCDHGB3	GI_14270495-A	1,13	2,88E-04	0,99	9,71E-01
MGC40574	GI_31341814-S	1,20	2,94E-04	1,03	8,97E-01
LOC375407	GI_37539713-S	1,18	2,97E-04	1,04	8,79E-01
ADIPOR2	GI_38261972-S	1,18	2,98E-04	0,94	8,57E-01
KIAA0953	GI_37546856-S	1,15	2,98E-04	0,95	7,55E-01
LOC400854	GI_42662088-S	1,26	3,03E-04	0,98	9,71E-01
KIAA1337	GI_18548545-S	1,16	3,04E-04	1,08	3,24E-01
LOC388300	GI_41150381-S	1,17	3,04E-04	1,00	9,87E-01

COL1A1	GI_14719826-S	1,13	3,07E-04	0,97	9,04E-01
FLJ11004	GI_31542664-S	1,23	3,07E-04	1,05	8,80E-01
WNT11	GI_17017973-S	1,22	3,07E-04	0,94	8,76E-01
GFER	GI_4885264-S	1,18	3,08E-04	0,99	9,71E-01
EDAR	GI_11641230-S	1,19	3,08E-04	1,06	7,55E-01
LOC375203	GI_37546908-S	1,22	3,08E-04	1,07	7,55E-01
LOC400629	GI_42661060-S	1,15	3,08E-04	1,00	9,71E-01
A4GALT	GI_34222135-S	1,20	3,12E-04	0,96	9,33E-01
CHODL	GI_20127635-S	1,15	3,14E-04	0,98	9,61E-01
CKLFSF1	GI_31563411-A	1,22	3,19E-04	1,01	9,71E-01
WDR5B	GI_42544246-S	1,23	3,19E-04	1,05	8,57E-01
CSAGE	GI_24234759-I	1,19	3,23E-04	1,04	8,57E-01
FLJ23235	GI_13376422-S	1,78	3,23E-04	1,05	9,71E-01
NCR2	GI_28395019-S	1,21	3,28E-04	1,01	9,71E-01
MADCAM1	GI_18780284-A	1,11	3,29E-04	0,95	7,56E-01
SNAI1	GI_18765740-S	1,16	3,47E-04	1,00	9,71E-01
YAP	GI_20986485-I	1,16	3,49E-04	1,01	9,71E-01
C1orf36	GI_34098969-S	1,16	3,54E-04	0,97	9,10E-01
MSH5	GI_26638661-I	1,22	3,54E-04	1,09	7,55E-01
ADAM2	GI_11497606-S	1,18	3,70E-04	1,03	9,32E-01
BASE	GI_28372506-S	1,19	3,70E-04	1,04	8,97E-01
COH1	GI_35493724-I	1,24	3,70E-04	1,04	8,98E-01
GPR135	GI_32261308-S	1,25	3,70E-04	1,04	9,04E-01
SLCO1B1	GI_19923779-S	1,19	3,70E-04	1,01	9,71E-01
SULF1	GI_29789063-S	1,17	3,70E-04	1,07	7,55E-01
TPM1	GI_34222222-S	1,18	3,70E-04	0,96	8,97E-01
LOC375512	GI_37552209-S	1,18	3,85E-04	0,99	9,71E-01
C20orf179	GI_30425403-S	1,26	3,87E-04	1,08	7,97E-01
HIRA	GI_21536484-S	1,33	3,87E-04	1,00	9,88E-01
ITGB1	GI_19743814-I	1,15	3,87E-04	1,00	9,73E-01
KIAA1409	GI_37545989-S	1,17	3,87E-04	1,01	9,71E-01
NKX2-2	GI_32307133-S	1,19	3,87E-04	1,00	9,88E-01
PCDHB8	GI_14195614-S	1,16	3,87E-04	0,99	9,71E-01
SLC23A1	GI_44680144-I	1,15	3,87E-04	1,02	9,34E-01
SOX7	GI_30581119-S	1,20	3,87E-04	1,05	8,79E-01
TMC3	GI_32469500-S	1,15	3,87E-04	1,03	8,79E-01

CNTN5	GI_28373127-I	1,17	3,91E-04	1,01	9,71E-01
AMACO	GI_38348303-S	1,11	4,04E-04	1,03	8,57E-01
ARHGAP6	GI_7382476-I	1,18	4,04E-04	1,03	9,09E-01
ELA2B	GI_7705647-S	1,15	4,04E-04	1,02	9,40E-01
PTP9Q22	GI_30089951-I	1,16	4,04E-04	1,04	8,57E-01
SLC14A1	GI_7706676-S	1,54	4,04E-04	1,04	9,58E-01
KIAA0211	GI_7661999-S	1,17	4,09E-04	0,99	9,71E-01
LOC138802	GI_37540519-S	1,16	4,10E-04	1,04	8,79E-01
LOC285647	GI_37550419-S	1,13	4,10E-04	1,00	9,71E-01
LOC400537	GI_42660771-S	1,43	4,12E-04	0,98	9,71E-01
C6orf61	GI_8923164-S	1,29	4,18E-04	1,02	9,71E-01
KIR3DS1	GI_7657280-S	1,17	4,18E-04	1,02	9,71E-01
LOC375472	GI_37550199-S	1,14	4,18E-04	1,00	9,87E-01
SLMAP	GI_6005873-S	1,11	4,18E-04	0,96	7,55E-01
LOC377129	GI_37550263-S	1,16	4,30E-04	1,05	7,55E-01
LOC339942	GI_42656826-S	1,20	4,32E-04	0,99	9,71E-01
POLR2J2	GI_21704273-I	1,14	4,32E-04	0,98	9,71E-01
LOC387982	GI_42660251-S	1,29	4,33E-04	0,98	9,71E-01
DKFZP566D193	GI_32698709-S	1,17	4,35E-04	0,97	9,61E-01
FLJ11164	GI_8922910-S	1,25	4,35E-04	1,02	9,71E-01
NTRK2	GI_21361305-S	1,26	4,35E-04	1,04	9,10E-01
ERBB2	GI_4758297-S	1,15	4,40E-04	0,95	8,57E-01
TCF20	GI_31652243-I	1,20	4,40E-04	0,99	9,71E-01
LOC377625	GI_37552685-S	1,17	4,44E-04	1,01	9,71E-01
LOC401135	GI_42657007-S	1,20	4,44E-04	0,92	7,97E-01
TPSG1	GI_6912727-S	1,18	4,63E-04	1,04	8,79E-01
LOC377120	GI_37550713-S	1,11	4,66E-04	0,97	7,97E-01
KIAA0133	GI_7661931-S	1,29	4,77E-04	1,01	9,71E-01
RANBP17	GI_22095364-S	1,18	4,88E-04	1,03	9,34E-01
GSG1	GI_24432099-I	1,20	4,90E-04	0,99	9,71E-01
CLCNKB	GI_4557474-S	1,24	4,99E-04	1,05	9,10E-01
PEPP-2	GI_14249118-S	1,16	4,99E-04	1,04	8,79E-01
C9orf41	GI_22748888-S	1,13	5,01E-04	0,98	9,57E-01
BPGM	GI_40353763-I	1,18	5,13E-04	0,98	9,34E-01
C14orf126	GI_18087836-S	1,34	5,13E-04	0,99	9,71E-01
DAPK2	GI_14670382-S	1,18	5,13E-04	1,01	9,71E-01

LOC115648	GI_21687265-S	1,24	5,13E-04	0,99	9,71E-01
RNPS1	GI_18379335-I	1,16	5,13E-04	0,98	9,71E-01
SNAP23	GI_18765728-I	1,24	5,13E-04	1,04	9,34E-01
TGFBR1	GI_4759225-S	1,20	5,15E-04	1,04	9,16E-01
FABP7	GI_36054052-S	1,16	5,26E-04	1,03	8,98E-01
ZNF533	GI_31542751-S	1,21	5,27E-04	1,02	9,71E-01
HACE1	GI_34222116-S	1,17	5,33E-04	1,02	9,61E-01
KIAA2028	GI_45120120-S	1,19	5,45E-04	1,01	9,71E-01
FISH	GI_7662103-S	1,15	5,47E-04	1,01	9,71E-01
LOC286103	GI_31341777-S	1,15	5,47E-04	0,97	8,79E-01
LOC389690	GI_41148533-S	1,19	5,48E-04	0,96	9,09E-01
UPK3A	GI_5902151-S	1,14	5,48E-04	1,00	9,71E-01
LGR7	GI_11056007-S	1,13	5,65E-04	1,01	9,71E-01
LOC388009	GI_42660347-S	1,17	5,79E-04	1,04	8,57E-01
LOC401620	GI_42662643-S	1,27	5,82E-04	1,03	9,34E-01
SHOX	GI_6031202-I	1,20	5,83E-04	1,06	8,57E-01
LOC121792	GI_17475183-S	1,17	5,84E-04	1,00	9,99E-01
FLJ39421	GI_31341837-S	1,15	5,91E-04	0,97	8,79E-01
FRMD1	GI_24432019-S	1,14	5,91E-04	0,99	9,71E-01
LOC122740	GI_37546044-S	1,16	5,95E-04	1,03	9,09E-01
VCL	GI_7669549-I	1,16	5,95E-04	1,01	9,71E-01
GRAP2	GI_19913386-S	1,27	5,97E-04	0,99	9,71E-01
C14orf29	GI_32528309-A	1,19	5,97E-04	0,97	9,04E-01
FKBP2	GI_17149843-A	1,17	5,97E-04	1,03	9,16E-01
LOC374386	GI_37541628-S	1,15	5,97E-04	1,00	9,88E-01
PCDH8	GI_6631101-I	1,16	6,10E-04	1,01	9,71E-01
APOBEC3F	GI_31083201-S	1,41	6,11E-04	1,00	9,88E-01
LOC400690	GI_42661599-S	1,41	6,11E-04	1,06	9,34E-01
AK5	GI_28144896-A	1,21	6,14E-04	1,05	8,57E-01
LOC376709	GI_37539612-S	1,18	6,19E-04	1,05	8,57E-01
BLR1	GI_14589868-I	1,17	6,34E-04	1,00	9,96E-01
FLJ36070	GI_32698969-S	1,19	6,39E-04	1,04	8,98E-01
LOC400051	GI_42659981-S	1,15	6,42E-04	0,92	7,55E-01
ACE	GI_23238215-I	1,13	6,53E-04	0,99	9,71E-01
LOC348938	GI_37549910-S	1,24	6,53E-04	1,01	9,71E-01
P2RX2	GI_28416918-I	1,15	6,53E-04	0,99	9,71E-01

KIAA1463	GI_39930390-S	1,29	6,67E-04	0,98	9,71E-01
SSH-3	GI_24586674-I	1,15	6,72E-04	1,01	9,71E-01
CR1L	GI_30146994-S	1,24	6,73E-04	1,04	8,97E-01
LOC402458	GI_42658205-S	1,18	6,73E-04	0,99	9,71E-01
RBM8A	GI_15812217-S	1,21	6,73E-04	1,00	9,92E-01
CYP4F11	GI_10863992-S	1,18	6,76E-04	0,96	8,57E-01
GTPBP5	GI_31563510-S	1,20	6,76E-04	1,01	9,71E-01
LOC400465	GI_42660608-S	1,14	6,80E-04	0,98	9,34E-01
LOC151760	GI_27478003-S	1,24	6,80E-04	1,03	9,34E-01
FLJ31121	GI_21389510-S	1,15	6,91E-04	0,93	7,97E-01
UNQ2446	GI_38348201-S	1,17	6,91E-04	0,99	9,71E-01
WFS1	GI_13376995-S	1,23	6,96E-04	0,98	9,71E-01
ZNF577	GI_14249251-S	1,39	7,00E-04	1,05	9,34E-01
CSRP2BP	GI_29570787-A	1,23	7,02E-04	1,04	9,34E-01
MYCBP	GI_8850230-S	1,19	7,07E-04	0,97	9,71E-01
EGFL8	GI_13449286-S	1,18	7,09E-04	0,97	9,09E-01
MGC35468	GI_23397531-S	1,12	7,09E-04	0,93	5,86E-01
PLA2G3	GI_7657125-S	1,12	7,19E-04	0,98	9,34E-01
KIAA1962	GI_27478473-S	1,10	7,24E-04	0,96	7,90E-01
LOC375328	GI_37550019-S	1,11	7,24E-04	1,01	9,71E-01
NOL1	GI_5453791-S	1,25	7,24E-04	1,04	9,34E-01
POP5	GI_38016925-I	1,17	7,24E-04	1,00	9,88E-01
SV2A	GI_7662271-S	1,20	7,24E-04	1,04	9,04E-01
NCK2	GI_4505346-S	1,21	7,25E-04	1,02	9,71E-01
CNGA2	GI_42718010-S	1,17	7,29E-04	0,99	9,71E-01
C6orf64	GI_8922866-S	1,26	7,35E-04	0,99	9,71E-01
PROL1	GI_10864034-S	1,17	7,35E-04	1,02	9,41E-01
FLJ12747	GI_37563751-S	1,16	7,39E-04	1,02	9,71E-01
NEK7	GI_19424131-S	1,18	7,49E-04	1,01	9,71E-01
RAX	GI_7305450-S	1,11	7,55E-04	0,98	9,34E-01
KCNJ14	GI_25777633-I	1,23	7,55E-04	0,99	9,71E-01
TRPM5	GI_24475627-S	1,13	7,57E-04	0,98	9,09E-01
IBSP	GI_13259536-S	1,13	7,62E-04	0,95	7,55E-01
MAP1D	GI_40385866-S	1,20	7,76E-04	0,98	9,71E-01
LOC120082	GI_37541482-S	1,12	7,80E-04	0,97	8,96E-01
LOC283162	GI_27499140-S	1,22	7,80E-04	1,04	8,97E-01

C21orf70	GI_17158022-S	1,26	7,82E-04	1,05	9,09E-01
FLJ35487	GI_28395052-S	1,14	7,90E-04	1,02	9,09E-01
FBXO9	GI_15812200-I	1,22	7,95E-04	0,99	9,71E-01
LOC402471	GI_42658248-S	1,16	7,96E-04	0,95	8,57E-01
UNQ541	GI_45504356-S	1,18	7,96E-04	0,96	8,97E-01
LCN7	GI_11545917-S	1,14	8,15E-04	0,96	8,57E-01
KIAA1272	GI_37556088-S	1,16	8,15E-04	1,00	9,88E-01
LOC375108	GI_39752678-S	1,16	8,17E-04	0,98	9,71E-01
TRIM50A	GI_31342404-S	1,15	8,17E-04	0,95	8,57E-01
EPAS1	GI_41327154-S	1,16	8,25E-04	1,03	9,09E-01
LOC165186	GI_40548323-S	1,11	8,28E-04	1,00	9,71E-01
LOC339541	GI_37547044-S	1,20	8,50E-04	1,03	9,34E-01
FLJ14100	GI_13376545-S	1,17	8,51E-04	1,01	9,71E-01
GABRA6	GI_4557606-S	1,20	8,51E-04	1,03	9,36E-01
TGM1	GI_4507474-S	1,30	8,91E-04	0,98	9,71E-01
IREM2	GI_31324529-S	1,10	8,94E-04	1,00	9,71E-01
C6orf212	GI_27482725-S	1,15	9,21E-04	0,99	9,71E-01
RFXDC1	GI_27734870-S	1,14	9,35E-04	1,00	9,71E-01
LOC131405	GI_37550025-S	1,11	9,41E-04	1,01	9,71E-01
RBPSUH	GI_34304114-I	1,12	9,69E-04	0,97	8,97E-01
UGT8	GI_40254470-S	1,15	9,87E-04	1,00	9,88E-01
FLJ10874	GI_8922727-S	1,12	1,00E-03	1,02	9,34E-01
KCNF1	GI_27436998-S	1,13	1,00E-03	1,01	9,71E-01
SFTPC	GI_42476334-S	1,10	1,05E-03	0,99	9,63E-01
LOC286436	GI_37546232-S	1,17	1,05E-03	1,04	8,97E-01
FLJ31882	GI_22748970-S	1,18	1,05E-03	1,01	9,71E-01
MGC10731	GI_34147392-S	1,16	1,06E-03	0,98	9,58E-01
CKM	GI_21536287-S	1,20	1,11E-03	0,98	9,71E-01
LOH12CR1	GI_31377627-S	1,12	1,11E-03	0,98	9,30E-01
MGC2562	GI_39725653-S	1,38	1,11E-03	0,97	9,71E-01
LOC377266	GI_37550443-S	1,19	1,11E-03	1,01	9,71E-01
NYD-SP18	GI_14211934-S	1,20	1,11E-03	1,02	9,71E-01
SYT8	GI_32528307-S	1,19	1,11E-03	0,99	9,71E-01
MGC48595	GI_42558269-S	1,12	1,12E-03	0,95	8,03E-01
KCNK4	GI_15718766-I	1,16	1,14E-03	1,02	9,71E-01
U1SNRNPBP	GI_31077202-A	1,28	1,14E-03	0,93	8,79E-01

SLC9A8	GI_41872702-S	1,18	1,15E-03	0,95	8,57E-01
CHX10	GI_34365782-S	1,14	1,16E-03	1,01	9,71E-01
SLC41A1	GI_40255193-S	1,11	1,18E-03	0,99	9,71E-01
FLJ45778	GI_38348367-S	1,13	1,18E-03	0,99	9,54E-01
caspr5	GI_20544138-I	1,13	1,19E-03	1,03	9,34E-01
CORT	GI_41327682-S	1,11	1,21E-03	0,98	9,34E-01
WFDC2	GI_18379361-A	1,16	1,21E-03	1,04	8,97E-01
OLIG2	GI_17978474-S	1,22	1,21E-03	0,98	9,71E-01
FBXL10	GI_32698762-S	1,13	1,23E-03	0,94	8,57E-01
COL13A1	GI_22027608-A	1,13	1,25E-03	0,96	8,57E-01
ADH4	GI_11496889-S	1,22	1,28E-03	0,98	9,71E-01
GNAQ	GI_40254461-S	1,12	1,29E-03	1,02	9,34E-01
MAFA	GI_42415526-S	1,14	1,31E-03	0,99	9,71E-01
CPA6	GI_24111254-S	1,16	1,31E-03	1,00	9,97E-01
TRIM10	GI_16519562-A	1,16	1,32E-03	1,02	9,71E-01
C6orf199	GI_34222175-S	1,18	1,32E-03	1,01	9,71E-01
FRZB	GI_38455387-S	1,10	1,32E-03	0,97	8,79E-01
LOC284943	GI_37564923-S	1,08	1,32E-03	0,97	8,79E-01
LOC400941	GI_42656495-S	1,12	1,32E-03	1,01	9,71E-01
FLJ25124	GI_24432064-S	1,23	1,33E-03	0,96	9,35E-01
ZNF541	GI_14149978-S	1,21	1,33E-03	1,01	9,71E-01
LOC375887	GI_37550975-S	1,12	1,34E-03	1,03	9,04E-01
NANOG	GI_13376297-S	1,14	1,34E-03	1,00	9,71E-01
NOS2A	GI_24041028-I	1,13	1,35E-03	0,96	8,79E-01
C9	GI_31542289-S	1,10	1,35E-03	0,98	9,57E-01
JPHL1	GI_33859758-S	1,13	1,37E-03	0,96	8,79E-01
JM4	GI_6005793-S	1,27	1,40E-03	0,98	9,71E-01
KRTAP20-2	GI_31791013-S	1,10	1,40E-03	1,00	9,71E-01
ANP32E	GI_23463320-S	1,13	1,40E-03	0,97	9,04E-01
OR2C3	GI_37620203-S	1,17	1,43E-03	1,02	9,71E-01
MGC2574	GI_13129103-S	1,16	1,43E-03	0,99	9,71E-01
S100A5	GI_4506766-S	1,15	1,44E-03	0,99	9,71E-01
MGC26963	GI_37059795-S	1,20	1,44E-03	1,01	9,71E-01
MYEF2	GI_33620746-S	1,11	1,45E-03	0,97	9,04E-01
RFT1	GI_31377635-S	1,10	1,45E-03	0,96	8,03E-01
ARHGEF17	GI_21361457-S	1,17	1,46E-03	1,00	9,73E-01

	LOC401644	GI_42659532-S	1,16	1,46E-03	1,00	9,75E-01
	LOC400650	GI_42661395-S	1,13	1,48E-03	0,99	9,71E-01
	PHF7	GI_27894288-I	1,16	1,50E-03	0,99	9,71E-01
	EIF2B4	GI_41281816-I	1,08	1,50E-03	0,96	8,57E-01
	HAS1	GI_4504338-S	1,18	1,51E-03	0,99	9,71E-01
	KNS2	GI_34916055-I	1,14	1,51E-03	0,95	8,57E-01
	HOXD9	GI_23397673-S	1,19	1,52E-03	1,02	9,71E-01
	TFAP2A	GI_4507440-S	1,20	1,54E-03	0,98	9,71E-01
	CBLN2	GI_40255263-S	1,14	1,54E-03	1,01	9,71E-01
	ARRDC3	GI_32698735-S	1,17	1,59E-03	1,03	9,34E-01
	LOC400360	GI_42660432-S	1,12	1,59E-03	0,99	9,71E-01
	MPDZ	GI_4505230-S	1,15	1,59E-03	1,02	9,71E-01
	LOC126295	GI_28315872-S	1,15	1,61E-03	1,01	9,71E-01
	KIAA0375	GI_27480863-S	1,19	1,62E-03	1,01	9,71E-01
	APOM	GI_22091451-S	1,15	1,65E-03	1,00	9,88E-01
	LOC374843	GI_37544988-S	1,26	1,65E-03	1,04	9,46E-01
	LOC375036	GI_37548664-S	1,15	1,65E-03	0,99	9,71E-01
	RANBP3	GI_4506408-I	1,15	1,65E-03	0,93	8,57E-01
	ZNF563	GI_21687236-S	1,15	1,65E-03	1,01	9,71E-01
	MGC2752	GI_33859747-S	1,41	1,67E-03	1,01	9,71E-01
	RASAL1	GI_4759025-S	1,22	1,70E-03	1,00	9,77E-01
	ZFP28	GI_22094124-S	1,08	1,72E-03	0,98	9,34E-01
	LOC375508	GI_37552191-S	1,17	1,72E-03	1,01	9,71E-01
	LOC286478	GI_27501414-S	1,14	1,76E-03	1,00	9,88E-01
	ZFP95	GI_21483180-I	1,15	1,76E-03	1,04	9,04E-01
	SPOCK	GI_15451924-S	1,12	1,77E-03	1,02	9,34E-01
	LOC392849	GI_41148408-S	1,16	1,80E-03	1,01	9,71E-01
	DKFZP564A022	GI_21361953-S	1,29	1,80E-03	0,94	8,97E-01
	LOC146489	GI_37541994-S	1,13	1,82E-03	0,99	9,71E-01
	C6orf76	GI_13376188-S	1,14	1,86E-03	0,99	9,71E-01
	LOC400681	GI_42662014-S	1,12	1,87E-03	0,99	9,71E-01
	GAL3ST-4	GI_31542834-S	1,34	1,87E-03	0,97	9,71E-01
	MGC34824	GI_27734974-S	1,16	1,87E-03	0,97	9,61E-01
cluster 2	FUT4	GI_4503810-S	0,77	1,03E-05	0,93	7,55E-01
	TSPYQ1	GI_13435134-S	0,83	1,03E-05	0,94	2,21E-01

K6IRS4	GI_31341312-S	0,84	4,40E-05	0,96	8,79E-01
AD-017	GI_8923854-I	0,71	6,07E-05	0,93	8,79E-01
ADAMTS18	GI_40806186-I	0,73	1,00E-04	0,88	7,13E-01
CGI-94	GI_7705808-S	0,78	1,00E-04	1,01	9,71E-01
MGC42157	GI_23397529-S	0,82	1,09E-04	0,96	8,79E-01
MTRR	GI_4505278-I	0,83	1,09E-04	0,97	8,97E-01
GIPR	GI_45545425-S	0,84	1,15E-04	1,01	9,71E-01
PSG3	GI_42476158-S	0,83	1,15E-04	0,98	9,34E-01
LOC201181	GI_37543738-S	0,87	1,31E-04	1,06	7,55E-01
PARD6B	GI_37555952-S	0,86	1,37E-04	0,99	9,71E-01
FZD7	GI_4503832-S	0,81	1,63E-04	0,98	9,71E-01
ING1	GI_38201666-I	0,76	1,75E-04	0,94	7,97E-01
MGC42638	GI_31341671-S	0,81	1,84E-04	0,96	8,79E-01
RNF6	GI_34305294-I	0,85	2,06E-04	0,99	9,71E-01
PCDH11Y	GI_14589941-A	0,86	2,26E-04	1,02	9,71E-01
AKAP28	GI_45433550-S	0,83	2,46E-04	0,91	2,21E-01
TPIP	GI_40549426-I	0,89	2,55E-04	1,06	7,55E-01
C14orf42	GI_37545960-S	0,80	2,76E-04	0,98	9,71E-01
EML1	GI_4758267-S	0,82	2,76E-04	0,96	8,98E-01
ADRA1A	GI_15451756-I	0,67	2,76E-04	0,93	9,34E-01
GPR38	GI_4504094-S	0,86	2,76E-04	0,98	9,34E-01
HSFY	GI_24211016-I	0,84	2,95E-04	0,95	7,55E-01
FOXA1	GI_24497500-S	0,77	2,98E-04	0,93	8,57E-01
LOC388528	GI_41150960-S	0,82	3,10E-04	0,95	8,79E-01
GAGE7	GI_11024640-S	0,87	3,19E-04	1,05	8,57E-01
KIAA1219	GI_34787408-S	0,84	3,19E-04	0,97	9,05E-01
GAGEB1	GI_9558760-S	0,89	3,28E-04	1,00	9,71E-01
CLDN3	GI_21536298-S	0,85	3,28E-04	0,99	9,71E-01
KCNE2	GI_27436977-S	0,86	3,54E-04	0,96	8,57E-01
DYRK2	GI_5922003-I	0,78	3,63E-04	0,94	8,96E-01
CEACAM7	GI_5901929-S	0,83	3,70E-04	0,98	9,61E-01
LOC51725	GI_7706492-S	0,80	3,70E-04	0,95	8,57E-01
RAB3B	GI_19923749-S	0,78	3,70E-04	1,00	9,88E-01
SLC26A8	GI_20336284-I	0,87	3,87E-04	1,01	9,71E-01
CDK10	GI_32528262-I	0,88	3,99E-04	1,02	9,71E-01
LOC254559	GI_37540955-S	0,85	4,17E-04	0,96	8,57E-01

LOC399921	GI_42659718-S	0,77	4,17E-04	0,92	7,86E-01
ZNF382	GI_31543988-S	0,80	4,17E-04	1,01	9,71E-01
CACNA1B	GI_4502522-S	0,78	4,32E-04	0,98	9,71E-01
CGI-72	GI_39653320-I	0,81	4,35E-04	0,94	7,86E-01
LOC389628	GI_42658875-S	0,86	4,35E-04	0,98	9,41E-01
PIWIL1	GI_34222090-S	0,87	4,35E-04	1,01	9,71E-01
MGC10981	GI_14249203-S	0,85	4,44E-04	1,07	7,55E-01
FLJ39441	GI_37541617-S	0,85	4,88E-04	0,96	8,57E-01
PCDH19	GI_37546220-S	0,85	4,88E-04	0,96	8,57E-01
GRIA3	GI_32528272-A	0,81	4,99E-04	0,99	9,71E-01
ADAM28	GI_7656862-I	0,84	5,10E-04	0,99	9,71E-01
TSC2	GI_10938006-I	0,84	5,13E-04	1,00	9,88E-01
AADAT	GI_33469971-I	0,85	5,26E-04	1,03	9,34E-01
KRT23	GI_27894338-I	0,82	5,35E-04	0,97	9,34E-01
PPL	GI_45439326-S	0,85	5,47E-04	0,96	8,57E-01
MAFF	GI_23111001-I	0,84	5,52E-04	0,98	9,34E-01
SYT14L	GI_32698758-S	0,82	5,81E-04	0,96	9,04E-01
TAC4	GI_26449156-S	0,83	5,91E-04	0,99	9,71E-01
TLX1	GI_19923318-S	0,84	5,91E-04	0,98	9,71E-01
LOC285777	GI_27483664-S	0,83	5,97E-04	1,03	8,97E-01
OR8B8	GI_28570169-S	0,81	6,14E-04	0,94	8,57E-01
MRPL15	GI_21265077-S	0,76	6,26E-04	1,03	9,71E-01
GUCA1B	GI_15887357-S	0,83	6,44E-04	0,96	9,04E-01
LOC402709	GI_42658723-S	0,81	6,58E-04	0,98	9,71E-01
LOC375598	GI_37538617-S	0,86	6,80E-04	0,99	9,71E-01
HOXD8	GI_23510373-S	0,86	6,91E-04	1,06	7,55E-01
LOC253970	GI_37545925-S	0,88	7,00E-04	1,06	7,97E-01
KIAA0319	GI_7662053-S	0,88	7,02E-04	1,02	9,58E-01
BDNF	GI_34106706-I	0,85	7,24E-04	1,05	8,57E-01
UGT1A1	GI_8850235-S	0,79	7,28E-04	0,94	8,57E-01
LOC400871	GI_42662343-S	0,83	7,29E-04	0,98	9,71E-01
LOC152831	GI_31343329-S	0,86	7,35E-04	0,99	9,71E-01
LOC374491	GI_37545353-S	0,87	7,35E-04	0,95	8,28E-01
IL1RL1	GI_27894323-A	0,80	7,47E-04	0,96	9,34E-01
FLJ25161	GI_40255264-S	0,79	7,48E-04	0,93	8,57E-01
CBWD2	GI_38016203-S	0,77	7,49E-04	0,98	9,71E-01

GDF1	GI_11641419-S	0,84	7,57E-04	1,02	9,71E-01
LOC376030	GI_37541166-S	0,84	7,57E-04	0,98	9,41E-01
RBMS1	GI_8400723-I	0,90	7,66E-04	1,06	7,55E-01
CYP4F2	GI_13435390-S	0,81	7,80E-04	1,01	9,71E-01
MSMB	GI_20302156-I	0,83	7,90E-04	1,03	9,34E-01
TOPK	GI_18490990-S	0,83	7,94E-04	1,03	9,46E-01
MGC20460	GI_16596683-S	0,86	8,12E-04	0,98	9,04E-01
DUSP10	GI_21536330-I	0,89	8,50E-04	0,99	9,71E-01
KLK11	GI_21618356-I	0,86	8,50E-04	1,00	9,81E-01
FLJ35961	GI_40255105-S	0,91	8,51E-04	1,02	8,79E-01
LOC115749	GI_37542922-S	0,87	8,85E-04	1,03	9,34E-01
NEUROD6	GI_27475984-S	0,87	8,94E-04	1,01	9,71E-01
LOC342293	GI_37541427-S	0,90	9,01E-04	1,00	9,71E-01
DCOHM	GI_29788788-S	0,90	9,13E-04	1,03	9,34E-01
ARMC4	GI_31657113-S	0,86	9,35E-04	1,05	8,79E-01
C14orf39	GI_28372512-S	0,84	9,35E-04	1,02	9,57E-01
LDB2	GI_4504970-S	0,81	9,69E-04	1,02	9,71E-01
LOC375614	GI_37538667-S	0,90	9,87E-04	1,03	8,97E-01
LOC283578	GI_37545972-S	0,88	9,88E-04	1,00	9,71E-01
KIAA0608	GI_37550783-S	0,90	9,88E-04	1,00	9,71E-01
C4orf6	GI_5031564-S	0,79	9,96E-04	0,97	9,49E-01
CREB3L2	GI_34740336-S	0,88	1,05E-03	1,00	9,77E-01
GOLPH2	GI_29550837-I	0,84	1,07E-03	1,05	9,04E-01
C9orf27	GI_10864018-S	0,88	1,09E-03	1,03	9,34E-01
LOC376707	GI_37539608-S	0,86	1,14E-03	0,98	9,34E-01
LOC387946	GI_41149918-S	0,88	1,16E-03	1,00	9,88E-01
LOC375045	GI_37548764-S	0,84	1,16E-03	0,98	9,71E-01
CNN3	GI_4502922-S	0,79	1,21E-03	1,10	8,57E-01
LOC375548	GI_37538337-S	0,88	1,23E-03	1,01	9,71E-01
SGCZ	GI_21040252-S	0,87	1,26E-03	1,02	9,71E-01
FTSJ1	GI_29029590-I	0,84	1,32E-03	0,95	8,57E-01
LOC125595	GI_37545854-S	0,83	1,40E-03	0,96	9,04E-01
KIAA1221	GI_7705301-S	0,87	1,46E-03	1,01	9,71E-01
CASC2	GI_31341470-S	0,74	1,50E-03	0,91	8,79E-01
AQP8	GI_45446751-S	0,85	1,50E-03	1,00	9,77E-01
ZNF28	GI_37552010-S	0,86	1,51E-03	0,98	9,71E-01

CD53	GI_21237756-S	0,78	1,55E-03	1,02	9,71E-01
ACTC	GI_10938011-S	0,87	1,59E-03	0,99	9,71E-01
HAPLN1	GI_40254458-S	0,92	1,59E-03	1,04	8,57E-01
LOC374989	GI_37540084-S	0,82	1,60E-03	0,96	9,34E-01
NT5C1B	GI_24308357-S	0,86	1,61E-03	1,00	9,96E-01
DMD	GI_5032290-I	0,84	1,64E-03	0,99	9,71E-01
INSM1	GI_44890063-S	0,85	1,64E-03	1,00	9,82E-01
SNX11	GI_23111027-I	0,83	1,67E-03	1,03	9,61E-01
PCDHGA10	GI_14196447-I	0,84	1,74E-03	0,97	9,57E-01
WFDC8	GI_31657106-I	0,91	1,74E-03	1,03	7,97E-01
HSPC157	GI_7661813-S	0,73	1,81E-03	1,10	8,57E-01
NYD-SP12	GI_15100157-S	0,84	1,82E-03	0,99	9,71E-01
